# The relationship of parental rearing behavior and resilience as well as psychological symptoms in a representative sample

**DOI:** 10.1186/1477-7525-12-95

**Published:** 2014-11-07

**Authors:** Katja Petrowski, Elmar Brähler, Markus Zenger

**Affiliations:** Department of Medical Psychology and Medical Sociology, University of Leipzig, Philipp-Rosenthal-Strasse 55, D-04103 Leipzig, Germany; Department Psychosomatic Medicine, University of Mainz, Untere Zahlbacher Straße 8, D-55131 Mainz, Germany

**Keywords:** Recalled parental rearing behavior, Resilience, Anxiety, Depression

## Abstract

**Background:**

Recalled parental rearing behavior is one of the factors influencing the strength of resilience. However, it is unclear whether resilience is a relatively stable personality trait or has a relational character whose protective strength changes over the course of life. Therefore, the association between recalled parental rearing and resilience as well as symptoms of anxiety and depression was investigated in respect to age and gender.

**Methods:**

N = 4,782 healthy subjects aged 14-92 (M = 48.1 years) were selected by the random-route sampling method. In this sample, an ultra-short form of the Recalled Parental Rearing Behavior Questionnaire, the German short version of the resilience scale, and two screening instruments for depression and anxiety (PHQ-2, GAD-2) were filled out. Structural equation modelling was used to analyze the data estimated with the maximum likelihood method approach.

**Results:**

The data revealed that rejection and punishment were clearly associated with lower resilience. Moreover, resilience had a strong connection to the symptoms of anxiety and depression. Resilience had the same quality of association in both men and women with respect to anxiety and depression. Furthermore, the effect of resilience did not vary across several age groups even though challenges may differ over a lifetime.

**Conclusion:**

Recalled parental rearing behavior such as rejection and punishment as well as control and overprotection exert a significant association on the strength of resilience. Resilience has an effect independent of gender and does not affect people of different age groups differently.

## Background

The concept of resilience was first used in (clinical) developmental psychology in long-term observations of high risk children [1–3]. Recently, the concept of resilience has been investigated in respect to vulnerability and resistance of adults in respect to psychopathology/mental health. Even though the theoretical framework of resilience in adults is just reformulated [4], the association of parental rearing and resilience–known from resilient children–and the age-and gender-specificity are still under investigation.

Some authors define resilience as a relatively stable protective factor in adults, others just focus the outcome pattern, and still others emphasize the relational character of resilience by types of responses [5]. Resilience as a stable **protective factor** enables or facilitates positive adaptation to stress exposure [6–8]. Protective factors include individual characteristics or capacities and features of the environment which are associated with positive adaptation in situations of adversity. The second concept of resilience broadly focuses on a good **outcome** in spite of serious threats to adaptation or well-being [9]. Still others particularly focus on the **types of responses** to stressors [10] based on a homeostasis/pre-stressor level which is disrupted by a threatening event. In this, a functioning in different life domains, emotional behavior and biological responses to stressors, and a return to a baseline functioning plays an important role [11]. On the one hand, adaptability depends on an ability to modulate and control one’s own affective mental state, on the other hand, it depends on the situational stressors [12, 13] by dropping ineffective coping strategies and restructuring emotions, cognitions as well as behavior [12–15]. This concept of resilience is commonly measured with the Resilience Scale by Wagnild and Young [15]. These different perspectives of protective factors, types of responses, and outcome seem to be integrated into a recent theoretical reformulation of the resilience concept by encompassing recovery, sustainability, and growth [4].

**Parental rearing as a protective factor for children** was investigated in the earliest resilience study (Kauai longitudinal study, [16]). Positive parental rearing behavior during a child’s formative years significantly counteracts the risk of a future manifestation of psychological disorders since it influences the development of resilience positively [17]. Due to the positive rearing behavior children develop self-confidence and feelings of self-efficacy as a basis for high resilience (see [18, 19]). This protective effect of parental rearing might be observed by positive associations between supportive parenting, high resilience, and good mental health. In contrast, children with controlling parenting showed low resilience [20] and those with harsh, cold, and authoritarian parental rearing showed signs of psychological disturbances and anxiety disorders [21, 22]. However, parental rearing was not included in the taxonomy of resilience resources in adults [5] even though it was mentioned in the theoretical reformulation of the concept of resilience in adults by Zauta et al. [4]. In addition, Swanson et al. [20] postulated that resilience mediates the relationship between parenting rearing and mental health. However, the mediating effect of resilience needs yet to be empirically investigated in adults.

In other studies on resilience it was investigated over the entire course of life to **old age** [23]. Researchers argue that resilience is neither determined congenitally nor is it achieved by employing the same coping skills throughout the course of life. As the situational load and the challenges change over the course of life, the corresponding coping skills must adapt by necessity as well ([23–26]). Leppert and Strauß [27] observed that resilience is lower and depression is higher in individuals aged 70 and older than in individuals aged 30 to 70. Independent of age, women showed less resilience than men (cross sectional data, [28]). However, Block and Block [12] postulated that resilience is a relatively stable dispositional ability showing stability in a longitudinal study up to the age of 30 with gender differences in the stability at a higher variability in women. Due to these diverse results based on the same resilience instrument, the concept of resilience needs to be investigated gender-specifically for the different phases of age. In addition, the protective effect of resilience during the different phases of age and the gender specificity would be of further interest. Respective empirical results have not yet been published for adults.

According to the literature, parental rearing is associated with a child’s resilience. Positive parental rearing behavior significantly counteracts the risk of a future manifestation of psychological disorders since it influences the development of resilience in a positive way [17, 29–32]. Positive associations between parental rearing, high resilience, and health [20] lead to the postulation that resilience mediates the relationship between parental rearing and health. However, the mediating effect of resilience needs to be investigated empirically. Therefore, the first general aim of the present study is (1) to replicate the effect of recalled parental rearing behavior on the magnitude of resilience and the psychological symptoms in adults (mediator model).

The second objective of the present study is to investigate whether recalled parental rearing has a gender-specific effect on resilience as well as on psychological symptoms in adults. Based on the literature, boys often report more experiences with structures, rules, autonomy, and emotional warmth in parental rearing than girls, which results in greater resilience in boys than in girls ([33–35]). In addition, girls who report experiences of chronic and intense disharmony in their family suffer more frequently from psychological problems than boys [34]. Therefore, possible differences regarding the efficacy of resilience in reference to psychological symptoms in men and women need to be taken into account. We hypothesize (2) that gender acts as a moderator in the relationship of resilience and psychological symptoms.

The third aim of this study is to investigate the age-specificity of resilience and the age-specific effect of resilience. Since resilience is theoretically defined as a personality trait [12] it cannot be less effective in old age. Rather, the self-reported age-specifically experienced parental rearing behavior [36] may be responsible for the age-specific effects of resilience among individuals below and above the age of 70. Therefore, we hypothesize (3) that there is a relationship of resilience to psychological symptoms that is not moderated by age.

## Methods

### Sample

In 2006, the USUMA (Unabhängiger Service für Umfragen, Methoden und Analysen) Berlin Polling Institute selected households and participants by random-route sampling [37]. Sixty-two percent of all contacted individuals filled out the questionnaire. Of these, only the final sample of *N* = 4,983 native German speakers with completed questionnaires was examined. Using information from the Federal Statistical Office, the final sample was approved to be nearly representative for the German residential population in 2006 with regard to age. The participants ranged in age from 14 to 94 (M = 48.08, SD = 17.91). Concerning the proportions of males and females in this sample, women were to some extent overrepresented in this sample (54% versus 51%; [38]). For further socio-demographic details of the sample see Table 1. All the participants volunteered and received a data protection declaration in agreement with the Helsinki Declaration.

**Table 1:** **Socio demographic variables of the sample**

	Total	Men	Women
	N = 5,036	N = 2,334	N = 2,702
	M (SD)	M (SD)	M (SD)
*Age, years*	48.37 (17.97)	48.11 (18.01)	48.59 (17.94)
*Age range*	14-92	14-92	14-92
*Relationship status*			
Married/living together	2,702 (53.7)	1,313 (56.3)	1,389 (51.4)
Married/separated	63 (1.3)	27 (1.2)	36 (1.3)
Single	1,220 (24.2)	695 (29.8)	525 (19.4)
Divorced	475 (9.4)	178 (7.6)	297 (11.0)
Widowed	576 (11.4)	121 (5.2)	455 (16.8)
*Education*			
No school diploma	56 (1.1)	20 (0.9)	36 (1.3)
Elementary school diploma	2,225 (44.2)	1,054 (45.2)	1,171 (43.3)
Middle school diploma	1,369 (27.2)	560 (24.0)	809 (29.9)
Community college	354 (7.0)	164 (7.0)	190 (7.0)
College diploma	146 (2.9)	70 (3.0)	76 (2.8)
High school diploma	384 (7.6)	180 (7.7)	204 (7.5)
Master diploma	328 (6.5)	187 (8.0)	141 (5.2)
School student	174 (3.5)	99 (4.2)	75 (2.8)

The study was approved in accordance with the ethics guidelines of the “German Professional Institutions for Social Research” (Arbeitskreis Deutscher Markt- und Sozialforschungsinstitute, Arbeitsgemeinschaft Sozialwissenschaftlicher Institute; Berufsverband Deutscher Markt- und Sozialforscher).

### Instruments

The Recalled Parental Rearing Behavior Questionnaire (Fragebogen zum erinnerten Elterlichen Erziehungsverhalten, FEE, [36, 39]) is the shortened German version of the Swedish questionnaire Egna Minnen av Barndoms Uppfostran (My memories of upbringing, EMBU; [39, 40]). It is a standardized questionnaire to assess three highly interrelated dimensions of recalled parental rearing behavior for each parent, i.e.: (1) Paternal/Maternal Rejection and Punishment assesses overly strict, discerning parental behavior and rejection which the child perceived as inappropriate. (2) Paternal/Maternal Emotional Warmth assesses affectionate, supportive, praising behavior without implying any unnecessary interference from the respective parent. (3) Paternal/Maternal Control and Overprotection assesses parental behavior which the child perceived as overly thoughtful, blaming, interfering, and constricting, thus reflecting a distinct orientation toward effort, performance, and high expectations by the respective parent.

The German short version, both the items and the three scales, showed satisfactory to good psychometric properties [41]. The internal consistency (Cronbach’s Alpha; α = .72 to. α = .89) indicated good reliability and high correspondence to the values obtained for the original Swedish long version of the EMBU from 14 countries (Cronbach’s Alpha; α = .72 to α = .90; N = 3.500; [42]). The short version of the FEE consists of a total of 12 items; the participants have to rate 6 for the mother and 6 for the father [41]. The evaluations were implemented on a four-point Likert scale in respect to how often they have experienced a certain situation in their childhood (1 = *No, never*, 2 = *yes, occasionally*, 3 = *yes, often*, 4 = *yes, always*). The three scales consist of two items each for mother and father. The scale values of the three scales range from 2 to 8. For the purpose of the present study, these scale values (one each for father and mother) were used as indicators of the three latent variables mentioned above. An example for an item is: “Have you been punished hard by your father, even for trifles (small offenses)?”

The German shortened version (RS-11, [43]) of the resilience scale by Wagnild and Young [15] was implemented. In this questionnaire resilience is conceptualized as the ability to use internal and external resources to cope with developmental tasks. The original version of the RS is comprised of 25 items. It is a standardized questionnaire to assess two factor-analytically derived dimensions: Personal Competence with 17 items and Acceptance of Self and Life with eight items. The scale Personal Competences assesses self-value, independence, containment, and persistence. The dimension Acceptance of Self and Life incorporates adaptability, tolerance, and flexibility.

The 11 items of the RS-11 have to be rated on a seven-point-Likert scale with 1 = I do not agree and 7 = I agree. High scale values on the scale represent high resilience in contrast to low scale values. Internal consistency (Cronbach’s Alpha) as reported for the original samples by Schumacher et al. [43] with α = .91 indicates very good reliability. The scale of the short version (RS-11) correlates very high with the scale of the original version (RS-25) with r = .95. An example for an item is: “I can usually look at a situation in a number of ways”.

Furthermore, the items of the resilience questionnaire were reduced to two parcels. The parcels were constructed according to Little et al. [44], whereupon the items with the highest factor loadings of the latent variable were allocated alternately to both parcels in descending order.

To assess the severity of depressive symptoms, the Patient Health Questionnaire-2 (PHQ-2) was used [45]. This validated ultra-short screening instrument was shown to have good psychometric properties and covers the two core symptoms of major depression: depressed mood and loss of interest, referring to the last two weeks. Response options range from 0 = “not at all” to 3 = “nearly every day”.

The General Anxiety Disorder-2 (GAD-2) was used to examine the intensity of anxiety symptoms [46]. The participants were asked how often they had been bothered by each of the two core symptoms of a generalized anxiety disorder during the previous two weeks (“nervousness, anxiety, or strain”; “not being able to stop or to control worries”). Answer alternatives range from 0 = “not at all” to 3 = “nearly every day”.

### Statistical procedure

To follow the first aim of this study–the mediating effect of resilience–a structural equation model approach was used. In this model, parental rearing behavior affects the amount of resilience, which in turn predicts the self-reported level of anxiety and depression.

**Figure 1:**
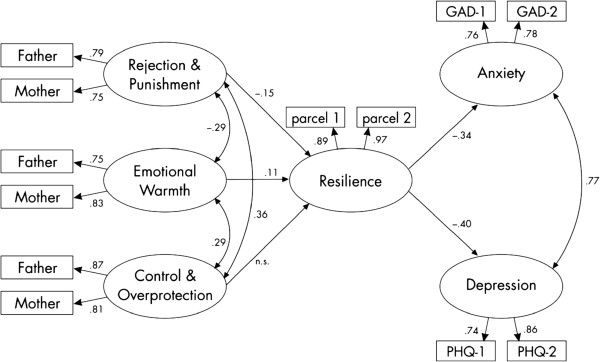
**Model of recalled parental rearing on resilience and on anxiety and depression (standardized values are depicted).** Note: all coefficients are significant with p<.00.1.

For each latent construct, two manifest indicators were taken into account. This was due to the focus of this study, namely the relationship between the examined constructs and not the detailed psychometric quality of the questionnaires, which can be assumed as good (with the exception of the original dimensionality of the RS-11). The final model is shown in Figure 1.

The statistical analyses were carried out with Amos 18 using the following model fit indices: the minimum discrepancy divided by its degrees of freedom (CMIN/DF); the root mean square error of approximation (RMSEA), the goodness-of-fit-index (GFI); the comparative-fit index (CFI); the normed-fit-index (NFI), and the Tucker-Lewis-Index (TLI). For a good model fit, the ratio CMIN/DF should be close to 3 or even smaller [47, 48], GFI, CFI and NFI should be higher than 0.95 for a good model fit [47] whereas values greater than .90 are usually interpreted as indicators for an acceptable fit [47–49]. Furthermore, RMSEA values smaller than 0.06 indicate a good model fit, and values smaller 0.08 still reflect an adequate fit [48–50]. The model was tested using covariance matrices, and it was estimated with the maximum likelihood method approach.

Following hypotheses (2) and (3), additional analyses were conducted to test the invariance of the model across gender and different age groups. At first, the model was computed for men and women separately. In a second step, we tested the measurement equivalency across men and women using multi-group analyses [51]. Measurement invariance was tested in six steps using (1) the unconstrained model, (2) followed by a metric invariant model (with equal measurement weights across groups), (3) a model with additionally equal structural weights, and (4) a model where the structural variances and co-variances were set equally in addition to the previous conditions. In the fifth and sixth step, further restrictions were defined with both (5) structural residuals and (6) measurement residuals were constrained to be equal across the groups. Based on the hierarchy of these nested and increasingly restrictive models, the models were then compared to each other [48]. Since Chi^2^ statistics have often been criticized for their sensitivity to the sample size, we focused on ΔCFI and ΔRMSEA as indicators in the comparison of models. Values smaller than .01 indicate the invariance of the models [52]. The same procedure was used to test for the invariance of the model for three age groups (<40 year-olds, 40-60 year-olds, >60 year-olds). These age groups were chosen with regard to the life course perspective as well as for statistical reasons. From the life course perspective the three groups can be differentiated into young adults who, for the most part, had finished their developmental tasks such as finding a job and starting a family, middle-aged, working adults in the phase of consolidation, and elderly adults in general facing the retirement phase. Furthermore, the comparison of substantial subgroups that do not differ too much in regard to sample size was intended and led to the age groups mentioned above.

## Results

Descriptive statistics of the study variables are shown in Table 2.

**Table 2:** **Descriptive statistics of the questionnaires used in this study (standard deviations in parentheses)**

Sum scores	All participants	Men	Women	T (p)	Age <40 y.	Age 40 -60 y.	Age >60 y.	F (p)	Post-hoc-Tests
Emotional warmth (father)	4.21 (1.50)	3.95 (1.42)	4.43 (1.54)	11.40 (<.001)	4.45 (1.61)	4.19 (1.46)	3.94 (1.37)	44.74 (<.001)	1 > 2 > 3
Emotional warmth (mother)	5.30 (1.43)	5.13 (1.39)	5.45 (1.44)	7.99 (<.001)	5.52 (1.43)	5.30 (1.41)	5.04 (1.39)	44.38 (<.001)	1 > 2 > 3
Control & overprotection (father)	3.26 (1.18)	3.28 (1.16)	3.25 (1.19)	1.03 (.301)	3.37 (1.24)	3.27 (1.15)	3.12 (1.12)	17.52 (<.001)	1 > 2 > 3
Control & overprotection (mother)	3.56 (1.30)	3.55 (1.28)	3.58 (1.31)	0.82 (.413)	3.68 (1.35)	3.60 (1.30)	3.39 (1.22)	21.33 (<.001)	1,2 > 3
Rejection & punishment (father)	2.74 (1.07)	2.85 (1.12)	2.64 (1.01)	7.08 (<.001)	2.60 (1.03)	2.75 (1.04)	2.87 (1.12)	24.91 (<.001)	1 < 2 < 3
Rejection & punishment (mother)	2.51 (0.91)	2.49 (0.88)	2.53 (0.94)	1.43 (.154)	2.45 (0.88)	2.53 (0.93)	2.57 (0.93)	7.34 (.001)	1 < 2,3
RS-11	59.61 (10.65)	59.97 (10.20)	59.29 (11.03)	2.31 (.021)	61.32 (10.41)	60.49 (10.09)	56.56 (10.95)	95.15 (<.001)	1,2 > 3
GAD-2	.82* (1.10)*	.70* (1.03)*	.93* (1.14)*	7.48 (<.001)	.75 (1.09)	.86 (1.10)	.87 (1.10)	6.11 (.002)	1 < 2,3
PHQ-2	.94* (1.20)*	.87* (1.17)*	1.00* (1.22)*	3.85 (<.001)	.86 (1.17)	.92 (1.17)	1.05 (1.27)	10.47 (<.001)	1,2 < 3

Note: * = values are first published in [53].

The first aim of the study was to replicate the relationship of the recalled parental rearing behavior to the levels of resilience and psychological symptoms. The hypothesized model depicted in Figure 1 fits the data rather well. Table 3 shows the fit indices of the entire structural equation model.

**Table 3:** **Summary of fit indices of the final structural equation model**

N	Chi ^2^(df)	p	CMIN/DF	CFI	GFI	RMSEA	TLI	NFI
5,036	923.225 (45)	<.001	20.516	.964	.972	.063	.947	.962

All but one fit measure indicated a good to acceptable model fit. The value of CMIN/DF indicated a relevant deviation between the data and the model since it should have been close to 3 for a correct model. On the other hand, this measurement is sensitive to the sample size. Thus, in case of a high sample size, even a small misspecification would lead to the rejection of the model. In accordance with Joereskog and Soerbom [54] we focused on the model fit indices described above, which are generally independent of the sample size.

Furthermore, all path coefficients shown in the model are significant with a p-value < .001. As shown in the model, the different dimensions of the FEE itself are weakly inter-correlated, and two of them predict the amount of resilience (standardized regression weights between .11 and -.15), which in turn predicts anxiety and depression negatively (-.34 and -.40, respectively). The standardized indirect effects of the three sub-dimensions of the FEE on anxiety and depression are very small (range: -.03 to .06). Based on the results, the assumption of a mediating role of resilience between the parental rearing behavior and psychological symptoms can be confirmed (hypothesis 1).

Additional analyses were conducted to test the equivalency of the model across gender and different age groups. The results are shown in Table 4.

**Table 4:** **Test for invariance across gender and age**

	N	Chi ^2^(df)	p for ΔChi ^2^	CMIN/DF	CFI	ΔCFI	RMSEA	ΔRMSEA
***Gender***								
Men	2,334	466.239 (45)		10.361	.961		.064	
Women	2,702	564.179 (45)		12.537	.961		.066	
***Multigroup analysis***								
Unconstrained model		1,030.418 (90)		11.449	.961		.046	
Equal measurement weights		1,041.934 (96)	.074	10.853	.961	<.001	.045	.001
Equal structural weights		1,049.651 (101)	.173	10.393	.961	<.001	.044	.001
Equal structural covariances		1,064.525 (107)	.021	9.949	.961	<.001	.042	.002
Equal structural residuals		1,089.146 (111)	<.001	9.812	.960	.001	.042	<.001
Equal measurement residuals		1,236.412 (123)	<.001	10.052	.954	.006	.043	.001
***Age groups***								
<40 years	1,704	320.550 (45)		7.123	.966		.060	
40-60 years	1,833	351.838 (45)		7.819	.964		.061	
>60 years	1,499	371.740 (45)		8.261	.957		.070	
***Multigroup analysis***								
Unconstrained model		1,044.135 (135)		7.734	.962		.037	
Equal measurement weights		1,070.816 (147)	.009	7.284	.962	<.001	.036	.001
Equal structural weights		1,095.570 (157)	.006	6.978	.961	.001	.035	.001
Equal structural covariances		1,189.183 (169)	<.001	7.037	.958	.003	.035	<.001
Equal structural residuals		1,225.187 (177)	<.001	6.922	.957	.001	.035	<.001
Equal measurement residuals		1,350.646 (201)	<.001	6.720	.952	.005	.034	.001

Note: All p-values of Chi^2^-Test between the subgroups were significant with p < .001.

*Abbreviations*: df degrees of freedom; CMIN/DF minimum discrepancy, divided by its degrees of freedom; CFI Comparative-Fit Index; RMSEA root mean square error of approximation.

As shown in Table 4, the multi-group analyses revealed the invariance of the models across both gender and three age groups as the differences of the fit indices between the unconstrained and the invariance model were smaller than .01. Thus, the structural equation model fits the empirical data very well for both men and women, and it is also invariant of age. Therefore, hypothesis (2) needs to be rejected whereas hypothesis (3) can be confirmed based on the results of these analyses.

## Discussion

Resilience as the capability of emotional resistance has been investigated in many studies in order to better understand the development of psychological symptoms (see review Dunkel, Schetter & Dolbier, [5]). The first aim of the current study was to replicate the relationship of recalled parental rearing and resilience as well as anxiety and depression in adults.

Our data showed that the hypothesized model fits the data rather well. Accordingly, recalled parental rearing does have a significant relationship with resilience and the level of anxiety and depression. This is in line with data showing that participants with positive parental rearing during childhood show high resilience and a lower future risk for a manifestation of psychological disorders (longitudinal data: [17, 32]). Also, the current data on adults showed an association between positive recalled parental rearing behavior such as emotional warmth and high resilience. Children who experienced an emotionally cold style of parental rearing in childhood more often suffer from several types of anxiety disorders [22, 55, 56]. The current data on adults showed that negative recalled parental rearing behavior such as rejection and punishment are negatively associated with resilience. Therefore, positive recalled parental rearing is associated positively and negative recalled parental rearing is associated negatively with resilience in adults. In adults, additional positive experiences due to social networking, social connectedness, social support, social cohesion, and close relationships might also have an impact and correct the negative parental rearing behavior on resilience over the life-span (see review Dunkel, Schetter and Dolbier, [5]).

The second objective of the study was to investigate whether there is a gender-specific association between recalled parental rearing, resilience, and the level of psychological symptoms in adults. We hypothesized (2) that resilience has a relationship with psychological symptoms dependent of gender. For children, the quality of experienced parental rearing and resilience differed by gender [34, 35]. In the present study, men showed slightly higher resilience than women. However, the multi-group analyses revealed an across-gender invariance of the models in a cross-sectional sample with participants between the ages of 14 and 94. For adults, the present data show similar across-gender associations between parental rearing and resilience as well as psychological symptoms. Longitudinal data would be required to draw conclusions on the gender-specific development of resilience as well as the psychological symptoms based on parental rearing since during adolescence there is a high rate of spontaneous remissions in the psychological problems [34, 57]. Therefore, the gender specificities of parental rearing, resilience, and psychological symptoms in children [17] may evolve during adolescence [57]. In adults, Leppert et al. [28] observed that women showed a lower resilience and fewer body symptoms independent of age. Based on the same instrument, in both studies this age-specific level of resilience would be replicated by our data as well. In addition, the effect of resilience is stable during the different phases of age.

Even though some authors define resilience as a relatively stable trait, others emphasize the relational character of resilience [28]. Therefore, in the present study, age specificity was investigated as a third objective. We hypothesized (3) that resilience has a relationship with psychological symptoms independent of age. The present data showed differences in the strength of resilience for the different phases of age. These results are in line with those by Leppert and Strauß [27]. They found that resilience as an intrapersonal resource throughout the course of life decreased with high age whereas depression increased. They argue that resilience loses part of its protective strength at this stage in life due to a decrease in autonomy [27]. This hypothesis is investigated in the present study as well. Herein, resilience is associated with symptoms of anxiety and depression independent of age. In contrast to Leppert et al. [28] recent studies show that older individuals do not display more psychological disorders or psychosocial stressors. In contrast to clinical intuition, the prevalence rate of psychological disorders is even lower than in younger individuals [58, 59]. One possible explanation might be the decoupling of the use of emotional suppression and psychological distress with age. When compared, older individuals show more use of suppression as an emotion regulation strategy than younger individuals [59]. Therefore, the present cross-sectional data point more towards resilience has a relatively stable effect as postulated by Block and Block [12] than towards resilience having a relational character [27]. Nevertheless, future longitudinal studies ought to investigate the trait character of resilience more thoroughly.

The strength of this study is its large representative sample and the statistical approach to the results. However, a large sample size might easily lead to small but significant correlation and regression coefficients, which is in parts underlined by the small coefficients found in the present study. In addition, the retrospective assessment of recalled parental rearing behavior represents a specific problem to assessing the actual parental rearing experienced during childhood or its subjective representation [60, 61]. The subjective representation may reflect the present mood, errors in the autobiographical memory (un-/conscious distortions), false memories, or idiosyncratic reconstructions of an individual’s personal history. However, the existing literature does not provide any consistent and conclusive data on the mood-congruent recall of relevant personal stimuli [60, 62–65] nor on the validity of retrospective data on parental rearing behavior [66]. Therefore, longitudinal studies with independent raters ought to be considered to validate parental rearing practices (see [67]). Unfortunately, in clinical practice, the child rearing behavior experienced by the patients can only be assessed retrospectively after the onset of the disorder. Nevertheless, even such belatedly obtained information can be of a certain help in the therapeutic process.

In sum, the present results clearly show that rejection and punishment are negatively associated with resilience. Furthermore, the lack of resilience is connected to the symptoms of anxiety and depression. However, resilience has a relationship to the symptoms of anxiety and depression in both men and women and its effect does not differ over time even though the challenges over a lifetime may change.
